# The micropolyurethane foam-coated Diagon/Gel^®^4Two implant in aesthetic and reconstructive breast surgery – 3-year results of an ongoing study

**DOI:** 10.3205/iprs000079

**Published:** 2015-12-21

**Authors:** Klaus E. Brunnert

**Affiliations:** 1 Klinik für Senologie, Osnabrück, Germany

**Keywords:** Diagon/gel®4Two implants, micropolyurethane foam-coating, synthetic meshes, acellular dermal matrices, lipofilling

## Abstract

**Background:** Breast implants are worldwide in use since 1962. Initially there were some problems with capsular contracture and the palpability of the rim of the implant. In 1968 this led to the introduction of the micropolyurethane foam-coating and then in 1970 to the first micropolyurethane foam-coated implant by F.A. Ashley. As a result of additional technical refinements in manufacturing this new implant design significantly reduced complications i.e. capsular contracture and implant rotation.

**Methods:** This study reports a single surgeon’s experience with aesthetic and reconstructive breast surgery, in primary and secondary cases with the sole use of micropolyurethane foam-coated Diagon/gel^®^4Two implants, partly in combination with the additional use of synthetic meshes, acellular dermal matrices and lipofilling. The trial is a prospective, single center cohort study designed to demonstrate the safety and effectiveness of the new implant design in primary and secondary aesthetic and reconstructive breast surgery. The reported data provide an interim report of the implantations performed from November 2010 to December 2013.

**Results:** 90 patients were admitted to the study with 152 implants. The majority of the implants (n=95, 62.5%) were used in reoperative cases for either oncological (n=52, 34.2%) or aesthetic reasons (n=43, 28.3%). The median age of the study cohort was 45 years; the median body mass index was 21; the median observation time is 41 months. There was a very low complication rate, both short term within 6 weeks after the implantation of the silicone gel implant and in the follow up in November 2015. There were no serious complications needing explantation, no capsular fibrosis or implant rotation or rupture so far. There were only 4 minor complications (1.97%). There was 1 local recurrence 4 years after skin and nipple sparing mastectomy.

**Conclusion:** The micropolyurethane foam-coated Diagon/gel^®^4Two implant is a very reliable silicone gel implant filled with two high cross-linked, cohesive and form stable gels. The study demonstrated a very high safety profile of the 4Two implant and its effectiveness in both primary and secondary reoperative aesthetic, oncological and reconstructive breast surgery. The study is ongoing, a longer follow-up will be needed to consolidate the data.

## Zusammenfassung

**Hintergrund:** Brustimplantate sind weltweit seit 1962 in Gebrauch. Von Beginn an gab es Probleme mit der Kapselfibrose und der Tastbarkeit der Implantatränder. 1968 führte dies zu der Einführung des mikropolyurethanbeschichteten Schaums zur Beschichtung der Implantate. 1970 wurde das erste mikropolyurethanschaumbeschichtete Implantat durch F.A. Ashley vorgestellt. Als Resultat späterer ergänzender technischer Verbesserungen in der Herstellung am neuen Implantatdesign konnte die Komplikationsrate z.B. von Kapselfibrose und Implantatrotation signifikant verringert werden.

**Methode:** Diese Studie berichtet über die Erfahrung mit dem ausschließlichen Gebrauch von mikropolyurethanbeschichteten Diagon/Gel^®^4Two-Implantaten für primäre und reoperative Fälle in der ästhetischen und rekonstruktiven Mammachirurgie, teilweise in Kombination mit dem zusätzlichen Gebrauch synthetischer Netze, azellulärer Dermis und Lipofilling. Die Studie ist prospektiv angelegt als Kohortenstudie in einem Zentrum, initiiert um die Sicherheit und Effektivität dieses neuen Implantatdesigns in der primären und reoperativen ästhetischen und rekonstruktiven Brustchirurgie zu demonstrieren. Die berichteten Ergebnisse stellen einen Zwischenbericht über Implantateinlagen dar, die von November 2010 bis Dezember 2013 durchgeführt wurden.

**Resultate:** 90 Patienten wurden in die Studie aufgenommen, mit der Einlage von 152 Implantaten. Die Mehrheit der Implantate (N=95, 62,5%) wurden in reoperativen Fällen aus entweder onkologischer (N=52, 34,2%) oder ästhetischer Indikation (N=43, 28,3%) eingesetzt. Das mediane Alter in der Studienkohorte war 45 Jahre, der mediane Body Mass Index 21; die mediane Beobachtungszeit ist 41 Monate. Die Komplikationsrate war sehr niedrig und zwar sowohl in der unmittelbaren postoperativen Zeit von 6 Wochen nach der Implantation des Silikongelimplantates als auch in der anschließenden Nachbeobachtungszeit bis November 2015. Es gab keine ernsten Komplikationen, die eine Explantation erforderten, bis zu diesem Zeitpunkt keine Kapselfibrosen oder Implantatrotationen oder Rupturen. Es gab nur 4 geringfügige Komplikationen (1,97%). Ferner 1 lokales Rezidiv 4 Jahre nach einer haut- und mamillensparenden Mastektomie.

**Zusammenfassung:** Das mikropolyurethanbeschichtete Diagon/Gel^®^4Two Implantat ist ein sehr zuverlässiges, Silikongel-gefülltes Implantat mit 2 hochvernetzten, kohäsiven und formstabilen Gelen. Die Studie demonstriert ein sehr hohes Sicherheitsprofil des 4Two Implantats und seine Effektivität sowohl in primärer wie auch reoperativer ästhetischer, onkologischer und rekonstruktiver Brustchirurgie. Die Studie wird fortgesetzt, zumal ein längeres Follow-Up benötigt wird, um die Daten zu konsolidieren.

## Introduction

In 1962 Thomas D. Cronin and Frank Gerow in collaboration with Dow Corning developed the first clinically usable silicone gel-filled breast implant [1]. From the beginning there were some problems with capsular contracture and the palpability of the rim of the implant. In 1968 this led to the introduction of the micropolyurethane foam-coating by William J. Pangman, and then in 1970 to the first micropolyurethane foam-coated implant by F.A. Ashley [2], [3]. As a result of additional technical refinements in manufacturing this new implant design significantly reduced complications i.e. capsular contracture and implant rotation [4], [5].

A modern silicone breast implant in use in aesthetic and reconstructive breast surgery requires above all functionality, i.e. stability, suitability and effectiveness. The aesthetic demands of our patients have increased. Due to the great variety of the phenotype of the individual breast contours we need individualized anatomical implants matching the different characteristics of the individual breast contour. The attributes of a modern silicone breast implant should therefore be the following: cohesive gel with a low oil content, a reliable barrier layer to prevent gel migration, form stability to reduce the occurrence of folds and wrinkling, different anatomical variants of shape and contour and notification of certain implant dimensions e.g. width, height, projection and the distance between the inframammary fold and the position of the nipple-areola-complex. 

**Figure 1:**
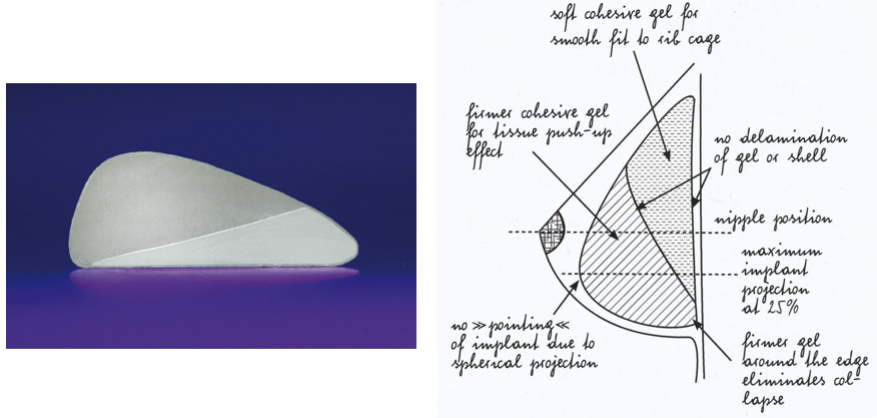
4Two implant

The micropolyurethane foam-coated Diagon/gel^®^4Two implant (Figure 1 (Fig. 1)) is a silicone gel implant, round and anatomical, with two high cross-linked, cohesive and form stable gels: a softer EasyFit Gel at the base and a firmer Shapar gel in the more frontal part. The softer gel and the slightly concave back of the implant provide the implant with an optimal footprint on the thoracic wall and lesser palpability of the edge of the implant base. The base of the Implant is either round or oval. The most characteristic difference of the implant type in contrast to other implant shapes is the different silhouette with the point of maximal projection at 25% of the entire vertical distance above the lower circumference of the implant instead of 35% which lifts the lower part of the breast together with the nipple-areola-complex. The firmer Shapar gel in the more frontal part of the implant smoothly regains its original foldless surface if it is compressed, which is especially required in case of a thin tissue coverage in order to prevent winkling.

The implants were introduced to the European market in 2010. 

## Patients and methods

This study reports a single surgeon’s experience in aesthetic and reconstructive breast surgery, in primary and secondary cases with the sole use of micropolyurethane-foam-coated Diagon/gel^®^4Two implants, partly in combination with the additional use of synthetic meshes, acellular dermal matrices and lipofilling. The trial is a prospective, single center cohort study designed to demonstrate the safety and effectiveness of the new implant design in primary and secondary aesthetic and reconstructive breast surgery. The implantation of the devices began in November 2010 and is still ongoing. The reported data provide an interim report of the implantations performed from November 2010 to December 2013. Patients were eligible for the study if they were at least 18 years of age, with either adequate tissue to cover the silicone implant, if the surgical intervention was a first line procedure for aesthetic or oncological reasons, or in case of a reoperative procedure the defects could be amended either with or without an ADM, synthetic mesh or lipofilling or a combination of the above mentioned. 

## Results

### Surgical characteristics

90 patients were admitted to the study with 152 implants. The majority of the implants (n=95, 62.5%) were used in reoperative cases for either oncological (n=52, 34.2%) or aesthetic reasons (n=43, 28.3%) (Table 1 (Tab. 1)).

**Figure 2:**
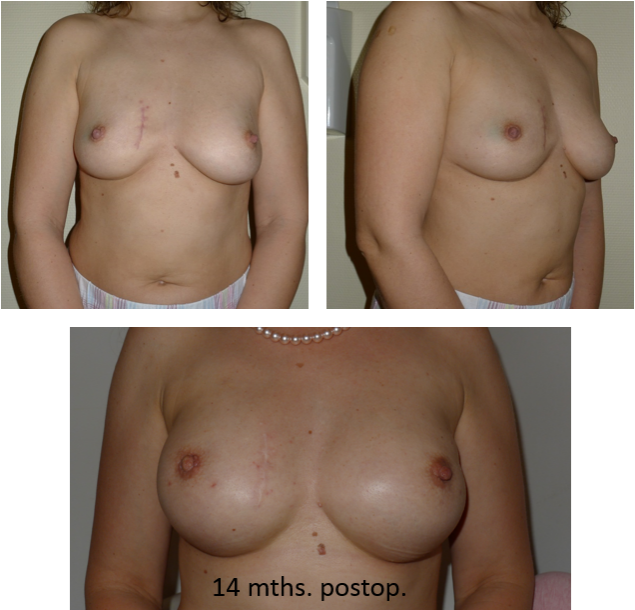
45-year-old pat., right breast: pT1c +pTis, left breast: pTis → bilateral nipple sparing MX, prepectoral MPS-4Two 320 ml bil. + coverage with synthetic mesh bil.

**Figure 3:**
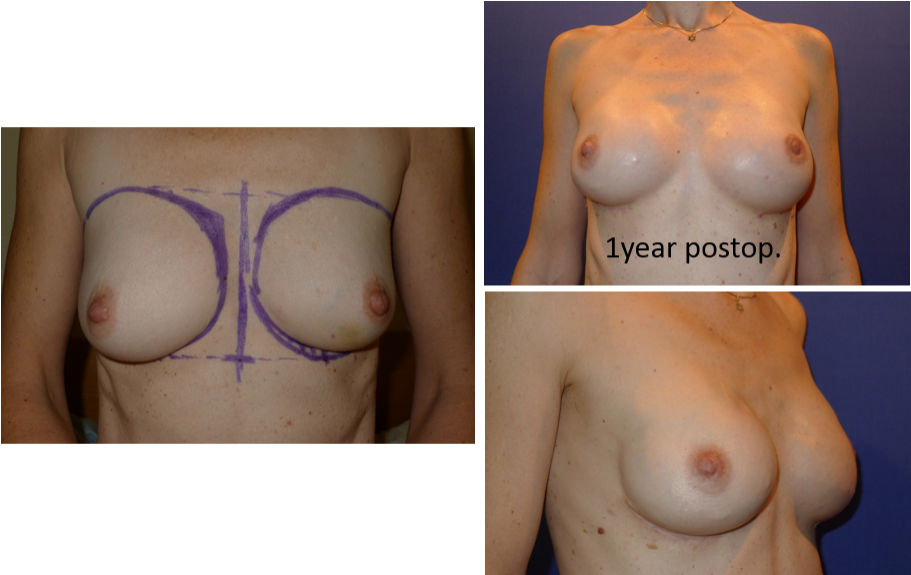
50-year-old pat., bil. nipple sparing mastectomy, 4Two + MPS, 235 ml, DCIS left, prophylaxis right, partially subpectoral + SurgiMend^®^ bil.

**Figure 4:**
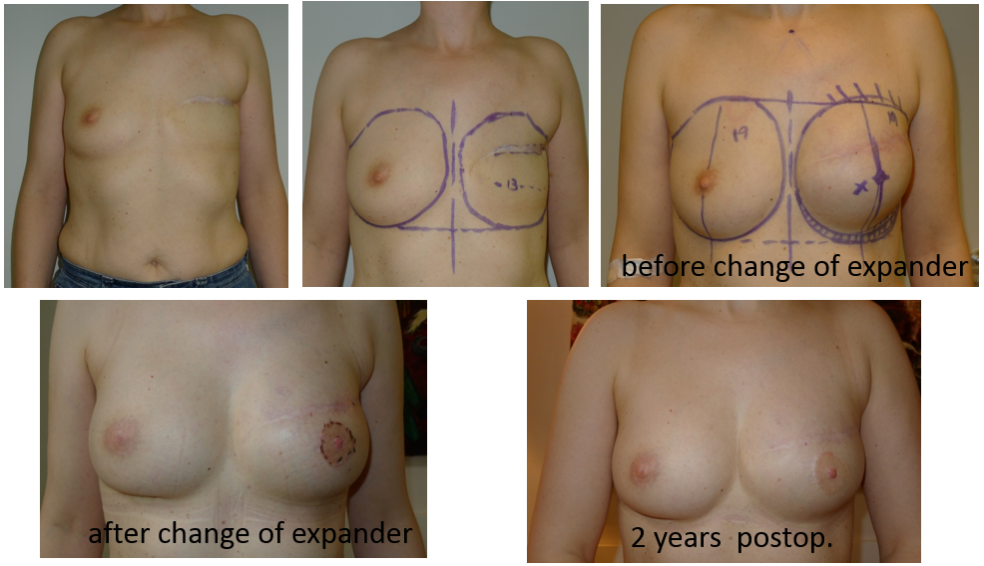
49-year-old pat., delayed breast reconstruction with staged expander/implant reconstruction, anatomical oval differential expander/4Two Implant 390 ml, anatomical, high profile, round base with synth. mesh fixation, lipofilling

29 devices (19%) were used for volume replacement in nipple/skin sparing mastectomies with immediate reconstruction in primary breast cancer, DCIS or BRCA 1 or 2 mutations (Figure 2 (Fig. 2) and Figure 3 (Fig. 3)). In the majority of cases, (n=23, 79.3%), the implant was placed in a partially subpectoral plane with the addition of ADM or synthetic mesh in 14 respectively 11 cases. Additional 15 implants (9.9%) were used for implantation to correct severe deformities after breast conserving therapy, 7 devices for primary augmentation mammoplasty and 8 devices for change of implants. Again, the majority of the implants (n=12, 80%) were placed partially in a subpectoral position; in 1 case with the addition of a synthetic mesh and in 2 cases with additional lipofilling (Figure 4 (Fig. 4)). 

71 implants in the study were used for aesthetic reasons, 28 (39.4%) with primary augmentation mammoplasty and 43 of 71 implants (60.6%) as reoperative cases with an exchange of implants due to problems after augmentation mammoplasty. Within the study group of aesthetic motivated surgery were 23 cases of congenital deformity (32.4%), either tubular breasts or Poland syndrome. 15 (65.2%) were reoperative cases, which needed in addition to an exchange of implant in 1 case an ADM, the insertion of a synthetic mesh in 2 cases and in 2 further cases lipofilling. 10 (43.5%) of the implants of the procedures to correct congenital deformities were placed prepectorally, 13 (56.5%) under the m. pectoralis major. Only 1 case of primary augmentation in the congenital deformity group needed lipofilling. All primary augmentations, excluding the congenital deformities, were performed via partially subpectoral implantation without the use of ADM, synthetic mesh or lipofilling. 

The median age of the study cohort was 45 years; the median body mass index was 21; the median observation time is 41 months.

**Figure 5:**
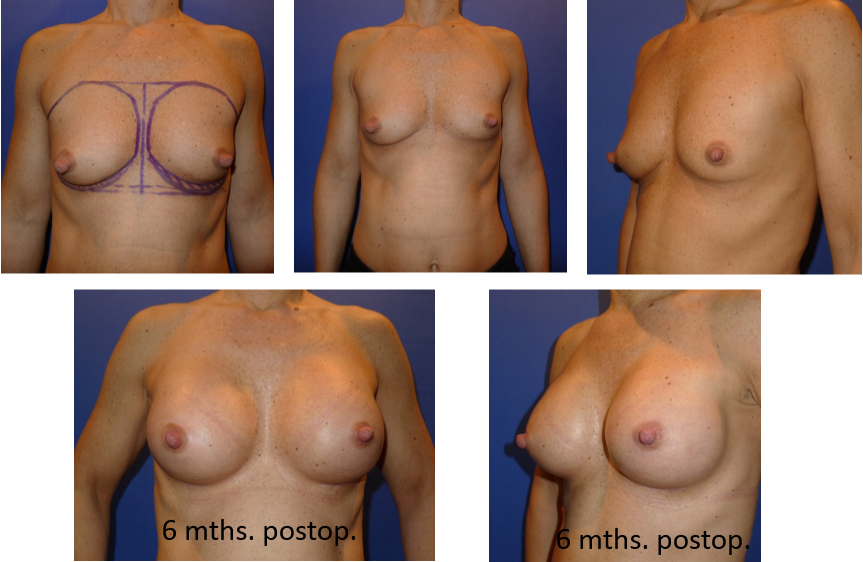
45-year-old pat., bil. augmentation mammoplasty, partially subpectoral, oval 4Two implants, high profile, 260 ml, MPS

**Figure 6:**
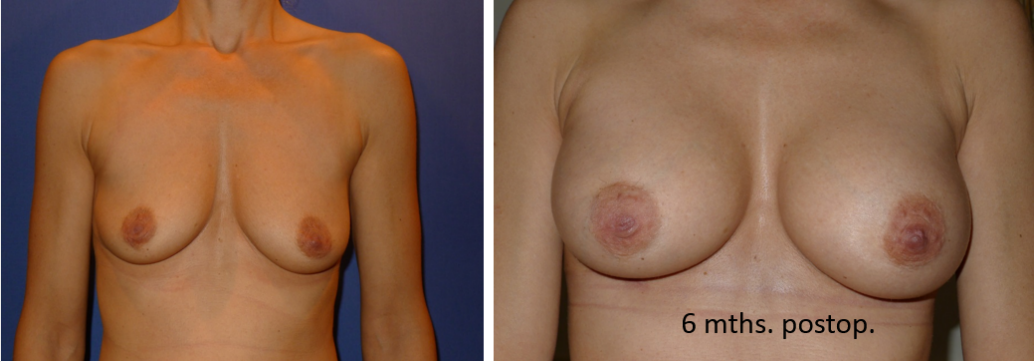
41-year-old pat., bil. augmentation mammoplasty, partially subpectoral, oval 4Two implants, high profile, 205 ml, MPS

**Figure 7:**
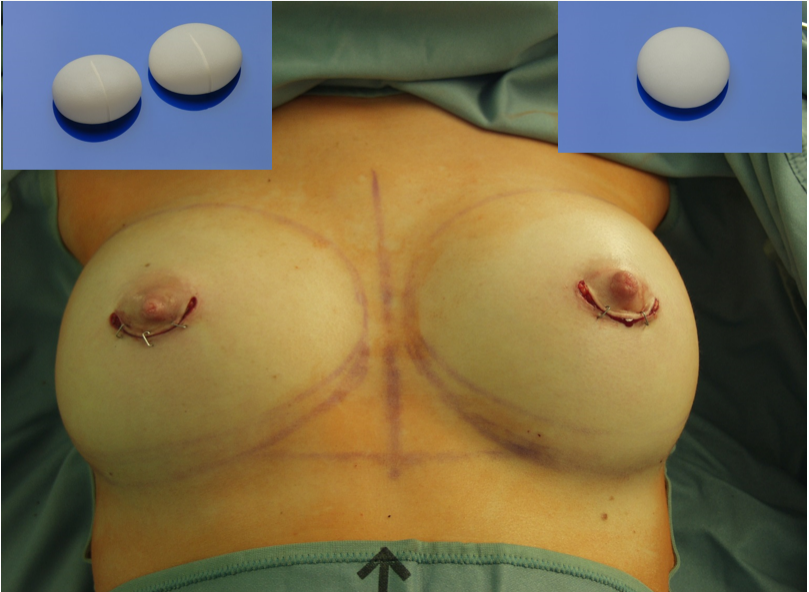
Making the right choice: anatomical vs. round implant. Right breast: anatomical oval implant, left breast: round implant.

See examples of aesthetic surgery in Figure 5 (Fig. 5), Figure 6 (Fig. 6) and Figure 7 (Fig. 7). 

### Safety

There was a very low complication rate, both short term within 6 weeks after the implantation of the silicone gel implant and in the follow up in November 2015. There were no serious complications needing explanation, no capsular fibrosis, no implant rotation and no rupture case so far. 2 patients developed complications needing minor surgery for successful treatment (Table 2 (Tab. 2)).

2 patients complained about palpable folds, one parasternally on one breast after bilateral prepectoral exchange of implant, the second at the upper curvature of the implant in partial subpectoral position with the problem solved by lipofilling. 1 patient with a history of radiation therapy and implant exchange due to capsular fibrosis developed postreoperatively a fistula into the implant pocket; the fistula was closed successfully with the use of an ADM and lipofilling. 1 patient developed postoperatively a larger seroma that disappeared after several needle aspirations within 6 weeks. There were no complications observed in primary aesthetic procedures and reoperations after congenital deformities. In total there were 4 minor complications (1.97%); 3 patients were successfully treated, 2 with minor surgical interventions. The patient with the parasternal fold decided to have no further treatment. 

There was 1 local recurrence 4 years after skin and nipple sparing mastectomy in an invasive lobular cancer. The local recurrence could be excised leaving the implant pocket intact with the patient ongoing the necessary further treatment.

## Conclusions

The micropolyurethane foam-coated Diagon/gel^®^4Two implant is a very reliable silicone gel implant filled with two high cross-linked, cohesive and form stable gels and a round or anatomical design. The study demonstrated a very high safety profile and its effectiveness in both primary and secondary reoperative aesthetic and oncological and reconstructive breast surgery. The firmer Shapar gel at the frontal layer of the implant provided a smoother, foldless and more formstable contour of the implant surface even in thinner tissue coverage. There was no interference between the polyurethane foam coverage of the implant and the use of acellular dermal matrices, synthetic mesh or the use of lipofilling. All patients were very satisfied with the aesthetic results. The study is ongoing, a longer follow-up will be needed to consolidate the data.

## Notes

### Competing interests

The author declares that he has no competing interests.

## Figures and Tables

**Table 1:**
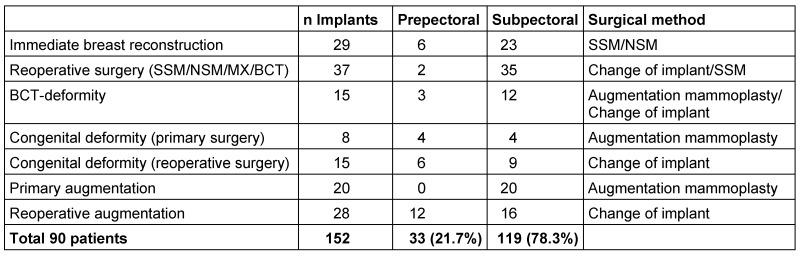
Surgical indications and characteristics

**Table 2:**
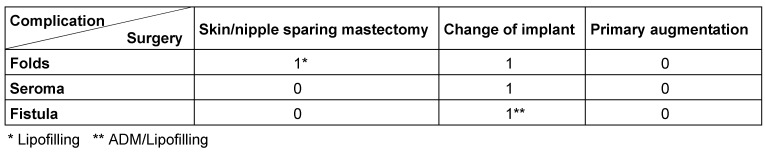
Complications
